# Nutritional and reproductive status affect amino acid appetite in house crickets *(Acheta domesticus)*

**DOI:** 10.3389/finsc.2023.1120413

**Published:** 2023-04-04

**Authors:** Ann Jane Tierney, Elizabeth Velazquez, Lauryn Johnson, Sarah Hiranandani, Meghan Pauly, Maya Souvignier

**Affiliations:** Neuroscience Program, Department of Psychological and Brain Sciences, Colgate University, Hamilton, NY, United States

**Keywords:** insect, cricket, amino acid, diet, feeding behavior

## Abstract

We examined amino acid appetite in the omnivorous house cricket (*Acheta domesticus*), a common model organism for both research and teaching. Our first experiment addressed the hypothesis that house crickets can discriminate between sucrose and essential amino acids (EAA), and that preference for the latter would be affected by prior feeding experience. To test this hypothesis, we compared feeding responses of juvenile and adult crickets following pre-feeding with sucrose or an essential amino acid mixture, predicting that sucrose-only pre-feeding would enhance subsequent intake of amino acids in a two-choice preference test. Based on previous studies, we also predicted that amino acid consumption would be enhanced in females compared to males, and in mated compared to virgin females. Hence we compared responses in male and female last instar nymphs, adult males, virgin females, mated females, and mated females allowed to lay eggs. The second experiment examined how extended periods of essential amino acid deprivation (48 h to 6 days) affected appetite for these nutrients in adult male and female insects. Finally, we examined growth and survival of juvenile and adult crickets fed a holidic diet lacking all amino acids and protein. Our results demonstrated that house crickets can distinguish EAA from sucrose and that consumption of the former is enhanced following sucrose-only pre-feeding. We also found sex and developmental differences, with juvenile and virgin females showing a greater preference for EAA than juvenile or adult males. Contrary to expectation, mated females preferred sucrose over EAA both prior to and after egg laying. We also found that the crickets of both sexes increased their intake of EAA when exposed to longer periods of deprivation, indicating that they engage in compensatory feeding on these nutrients. Finally, as expected we found that growth was severely limited in juveniles fed a diet lacking all amino acids, but adults and many juveniles survived for 30 days on this diet.

## Introduction

Omnivorous animals forage in complex and heterogenous food landscapes and must choose the correct mix of nutrients for growth and fitness. Many insects have evolved the ability to make such choices and will select an array of foods that offer the correct balance of essential nutrients. Researchers have used artificial diets to examine the regulation of macronutrients, especially protein and carbohydrates, in a number of diverse species (1). These studies have demonstrated that insects feed selectively when offered two foods which differ in protein:carbohydrate ratios, causing intake of each nutrient to match a ratio that promotes species-specific fitness (2–9). Insects also regulate the amount of food consumed and adjust their intake depending on the nutrient density of the food. For example, grasshoppers compensate behaviorally for nitrogen dilute diets by increasing the number and duration of feeding bouts (10); bumble bees decrease consumption of pollen as the concentration of protein increases (11). An additional key finding is that, following protein or carbohydrate deprivation, insects subsequently engage in compensatory feeding on the limited substance to restore nutrient balance. The compensatory behavior can occur following days of deprivation or just a single meal that lacks a macronutrient (12–16).

In addition to macronutrient selection, insects can distinguish other substances, including micronutrients and free amino acids. Amino acids are important since they may signal the presence of protein and are necessary for numerous biochemical functions, including neurotransmitter biosynthesis. The ability of insects to perceive free amino acids and self-select food based on this perception has been studied in *Drosophila* adults and larvae (17–19) and nectar-feeding insects such as bees, ants, and mosquitoes (20–25). These studies demonstrate that an amino acid and sugar mix may be preferred over sugar alone, and that individual amino acids vary in their ability to stimulate feeding. Studies have also demonstrated that diets lacking essential amino acids (EAAs) can cause rejection of the unbalanced food (26) and enhance attraction to and feeding on amino acid mixtures or protein (17, 27). Lack of even a single EAA can induce this compensatory feeding on amino acid mixtures or protein (24, 28). Adaptive changes in amino acid intake can involve changes in search behavior to locate sources of amino acids or changes in sensory neuron responses to favor amino acid feeding (1, 29).

In addition to nutritional state, responses to protein, carbohydrate, and other nutrients vary with developmental stage, sex, and mating status. For example, Cohen et al. (3) found that carbohydrate consumption varied as cockroach larvae developed through successive instars, with intake highest directly following molting. Carbohydrate intake also shifted in mealworm larvae becoming greater in later compared to earlier instars (9). In *Drosophila*, larvae chose to linger on yeast patches (30) whereas well-nourished adult *Drosophila* fed preferentially on sucrose. However, compared to males, female *Drosophila* show a greater preference for feeding on yeast, a source of protein (18). Likewise adult crickets of both sexes prefer carbohydrate over protein (31, 32), but females consume more protein than males (31). The preference for protein was markedly enhanced in *Drosophila* females after mating, a response attributed to an elevated need for protein to produce eggs (15, 33). In the cricket *Gryllus bimaculatus*, protein consumption was also greater in mated compared to virgin females (34). Because the dietary switch occurred after the female crickets laid eggs, Tsukamoto et al. suggest that the enhanced protein appetite reflected the need to produce additional eggs for future episodes of mating and egg laying.

The house cricket (*Acheta domesticus*) is a common model organism for both research and teaching and is also important commercially as pet food and more recently as a source of protein for human consumption (35). Like many cricket species, *A. domesticus* is omnivorous, consuming foods that vary greatly in nutritional content across space and time. These feeding habits predict that crickets will be tolerant of temporary nutritional imbalances and able to address imbalances by compensatory feeding when needed nutrients become available (14). Compensatory feeding on macronutrients has been shown in several cricket species, but the ability to detect and self-select amino acids has not yet been studied. The quantity of amino acids consumed affects growth, reproduction, and longevity in insects and, as in mammals, certain amino acids are essential and must be acquired from the diet. We designed experiments to examine the ability of house crickets to detect EAA, and investigated how feeding decisions are affected by nutritional state, age, and sex.

Our first experiment addressed the hypothesis that house crickets can discriminate between sucrose and EAA, and that preference for the latter would be enhanced following a meal containing only sucrose. In this experiment, we used a two-choice preference test to compare food intake and feeding behavior of crickets following pre-feeding with sucrose or EAA. Previous studies have demonstrated that appetite for carbohydrates and protein can vary significantly in insects depending on age and sex. To determine if such variation occurs in house crickets, we tested males and females separately and compared responses in last instar nymphs and adult animals. Previous studies have also reported that female reproductive status affects protein appetite, and hence the first experiment included two additional groups: mated females, and mated females allowed to lay eggs. For the latter comparisons, we predicted that amino acid appetite would be enhanced in females compared to males, and in mated compared to virgin females. In our second experiment, we used extended periods of EAA deprivation to determine if crickets would subsequently engage in compensatory feeding on an amino acid mixture. Finally, to further understand cricket motivation for amino acid consumption, we examined growth and survival of juvenile and adult crickets fed a holidic diet lacking all amino acids and protein.

## Materials and methods

### Insects and diets

Juvenile and adult *A. domesticus* were acquired from laboratory-reared colonies originally established using animals from commercial suppliers. They were maintained at 27° C, 12:12 light:dark with ad libitum access to a standard food (Mazuri Cricket Diet) and water. To obtain virgins, females were removed from the colony before molting to adults and housed in female-only tanks (45 x 25 x 25 cm high) prior to testing. Males used in experiments were taken from the mixed sex colonies. For food preference tests, crickets were tested in same sex groups of five in clear plastic containers (19 x 28.5 x 23.5 cm high) equipped with an egg carton shelter and water. For survival tests, crickets were held in the same plastic containers with three adults or ten 4^th^ instar juveniles per container.

Test diets for food preference tests consisted of pure 0.5 M sucrose, a mixture of 10 essential L-amino acids (lysine, tryptophan, histidine, phenylalanine, leucine, isoleucine, threonine, methionine, valine, and arginine), and a mixture of 0.5 M sucrose plus 10 EAA. We opted to incorporate chemicals into agar gel which kept nutrients dissolved and prevented food from being scattered or carried by the crickets during feeding. Pure sucrose was made by combining distilled water with 2% agar and adding sucrose to form a 0.5 M solution. For the amino acid mixture, we followed other protocols by using an equimolar mixture with amino acids each at 10 mM dissolved in distilled water and 2% agar (22, 36, 37, Ignall et al., 2021). For the sucrose/amino acid mixture, amino acids were added to the liquid 0.5 M sucrose agar solution. The latter mixture was used during pre-feeding to ensure that insects seeking sucrose would necessarily ingest amino acids as well.

To test the survival of crickets fed EAA-free food, holidic diets were created following the recipe developed for other orthopterans (6, 38). The complete holidic diet contained 2.4% Wesson salt mix, 0.5% linoleic acid, 0.5% cholesterol, 0.2% Vanderzant vitamin mix, 10 EAA each at 5 mM, 0.5 M sucrose, and 2% agar. The amino acid free diet included all chemicals except the 10 amino acids, and a control diet contained 20% standard cricket food mixed with 2% agar. After chemicals were completely dissolved, solutions were cooled to 55°C, poured into 100 x 15 mm petri dishes, and refrigerated until use. Chemicals were acquired from the following sources: Wesson salt mix (Fisher Scientific, Pittsburg PA), Vanderzant vitamin mix (Frontier Scientific Inc., Logan, UT), L-threonine and L-leucine (Cayman Chemical Co., Ann Arbor, MI), all other chemicals (Sigma- Aldrich Inc., St. Louis, MO).

### Experiment 1: Pre-feeding with sucrose or EAA and subsequent food preference

**Figure 1:**
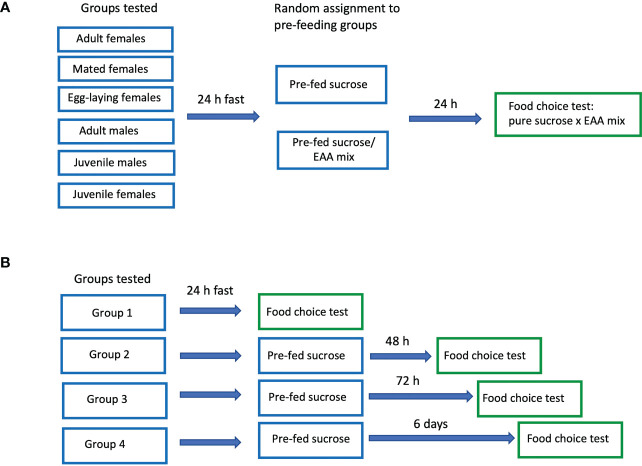
Schematic diagram of protocols used in Experiments 1 **(A)** and 2 **(B)**.

Experiment 1 examined the effects of pre-feeding with sucrose or EAA on subsequent preference for the two foods. The following groups were tested: juveniles (last instar nymphs), adult males, adult virgin females, adult mated females, and adult mated females allowed to lay eggs. For each of the five groups, 16-20 containers were tested, half pre-fed sucrose and half pre-fed the sucrose/EAA mix (80-100 crickets/group; 530 crickets total; each cricket participated in only one test). Crickets were placed in containers and fasted (no food available) for 24 h after which they received food consisting of pure 0.5 M sucrose or 0.5 M sucrose/EAA mix in agar. Food was placed on plastic petri dish (15 mm) lids and set in the tanks for four hours. 24 h later crickets participated in a two-choice preference test in which they received two types of food: pure 0.5 M sucrose and the pure EAA food described above (**Figure 1A**). Each food type for pre-feeding or choice tests was provided by cutting uniform disks from agar plates, each disk weighing approximately 425 mg and measuring 9 mm in diameter and 5 mm high. In choice tests, the two disks were each placed on a plastic petri dish lid and set adjacent to each other in the tank. As in pre-feeding, crickets were allowed access to the food for four hours. To determine the exact amount of food consumed, each disk was weighed to the nearest 0.1 mg before and after the trial period. If no food of either type was consumed, the tank was eliminated and the experiment replicated with additional crickets to yield the full data set. To correct for evaporation, control disks were treated in an identical manner, but held in containers without crickets for the 4 h period. The amount of food lost to evaporation was converted to percent values which were then subtracted from the test disks after each experiment. All tests were video-taped for the entire four hours and adult cohorts (virgin females, mated females, egg laying females, and adult males pre-fed EAA and sucrose) were subsequently watched by trained observers blind to the treatment group and contents of each dish. The number of visits and the total amount of time crickets spent feeding on each dish were quantified for the first hour of each trial. A visit was recorded when a cricket’s mouthparts contacted food for at least 2 seconds, and time feeding was recorded when a cricket’s mouthparts were in contact with the food. Visits and time feeding by all five crickets were added giving an overall score for each measurement for each tank.

Mated females were acquired by introducing males to tanks housing only adult virgin female crickets (the number of each sex were equal) and allowing the animals to interact freely for 48 h. Under our conditions, males began courtship singing within 15 min of exposure to females and matings were observed within two hours. To acquire females allowed to lay eggs, males were removed after 48 h and a plastic dish (12 cm diameter x 6 cm high) of moistened soil was placed in the container housing the mated females. Females began laying eggs in the soil within 30 minutes and they had access to the soil containers for 24 h. Directly after the egg laying period, these females, mated females, and virgin females were placed in test containers, fasted, pre-fed either pure 0.5 M sucrose or 0.5 M sucrose/all EAA mix, and tested in the two-choice preference assay as described above.

### Experiment 2: Extended amino acid deprivation and subsequent food preference

In experiment 2, adult male and virgin female crickets were tested under four different conditions: Group 1, 24 h EAA deprivation; Group 2, 48 h EAA deprivation; Group 3, 72 h EAA deprivation, and Group 4, six days EAA deprivation (**Figure 1B**). For all experiments, crickets were placed in test containers and fasted for 24 h. After this period, Group 1 received a choice of two food disks: pure 0.5 M sucrose and the mixture of 10 EAA. The food was measured and presented in the manner described above. For Groups 2, 3, and 4 sucrose pre-feeding only was used prior to the two-choice preference test. In Group 2, crickets were supplied with a disk of pure 0.5 M sucrose after the initial 24 h fast and allowed to feed for four hours. The following day, they were given a choice of pure sucrose or pure EAA as described for Group 1. Likewise Groups 3 and 4 received pure sucrose disks each day for 2 and 5 days respectively, and then tested the following day with the sucrose/EAA choice as described for Group 1. Each Group consisted of 16-18 containers (80-90 crickets/group) with equal numbers of males and females. All tests were videotaped for the entire four-hour period.

### Experiment 3: Growth and survival in amino acid-deprived crickets

In experiment 3, survival of adult and juvenile crickets over a 30-day period was examined by supplying insects with one of three diets described above: control, holidic with all EAA, and holidic with no EAA. Crickets were housed in plastic tanks (19 x 28.5 x 23.5 cm high) equipped with an egg carton shelter and water. Each tank was supplied with a fresh disk of the assigned food every day. Juvenile crickets (N = 180, 60 per group) were in the 4^th^ instar at the start of the experiment and were housed 10 per tank. Tanks were checked twice a day and dead bodies were immediately removed. This reduced, but did not completely eliminate, cannibalism among juveniles. Consequently, an additional experiment was conducted with similar 4^th^ instar juveniles in isolate containers exposed to the control or no EAA diet (N = 20, 10 per group). To monitor growth, living juveniles were weighed at the start of the experiment and at the end of the 30-day feeding period. Adult crickets (N = 54, 18 per group) were separated by sex and housed three insects per tank.

### Statistical analyses

Statistical analyses were conducted using SPSS with statistical significance set at alpha = 0.05. Preliminary analyses of food intake and behavior revealed significant deviations from normality and homogeneity of variance for data in some groups (Shapiro-Wilks test, P < 0.05; Levene’s test. P < 0.05) and several data sets included outliers. Hence we used nonparametric tests to evaluate differences within and among groups. For experiment 1, we initially focused on nutrient choice within each group and used Wilcoxon signed-rank tests to compare food consumption and feeding behavior following pre-feeding with sucrose or EAA. To compare EAA intake among groups, we used a non-parametric analysis of variance (Kruskal-Wallis test) and, where appropriate, conducted multiple comparisons with Dunn-Bonferroni tests. For experiment 2, we also used Kruskal-Wallis and *post-hoc* Dunn-Bonferroni tests to compare sucrose and EAA intake in male and female crickets subjected to four different periods of EAA deprivation. For experiment 3, Kaplan-Meier log-ranked tests were used to compare survival distributions in groups which received control diet or holidic diets with and without EAA.

## Results

**Figure 2:**
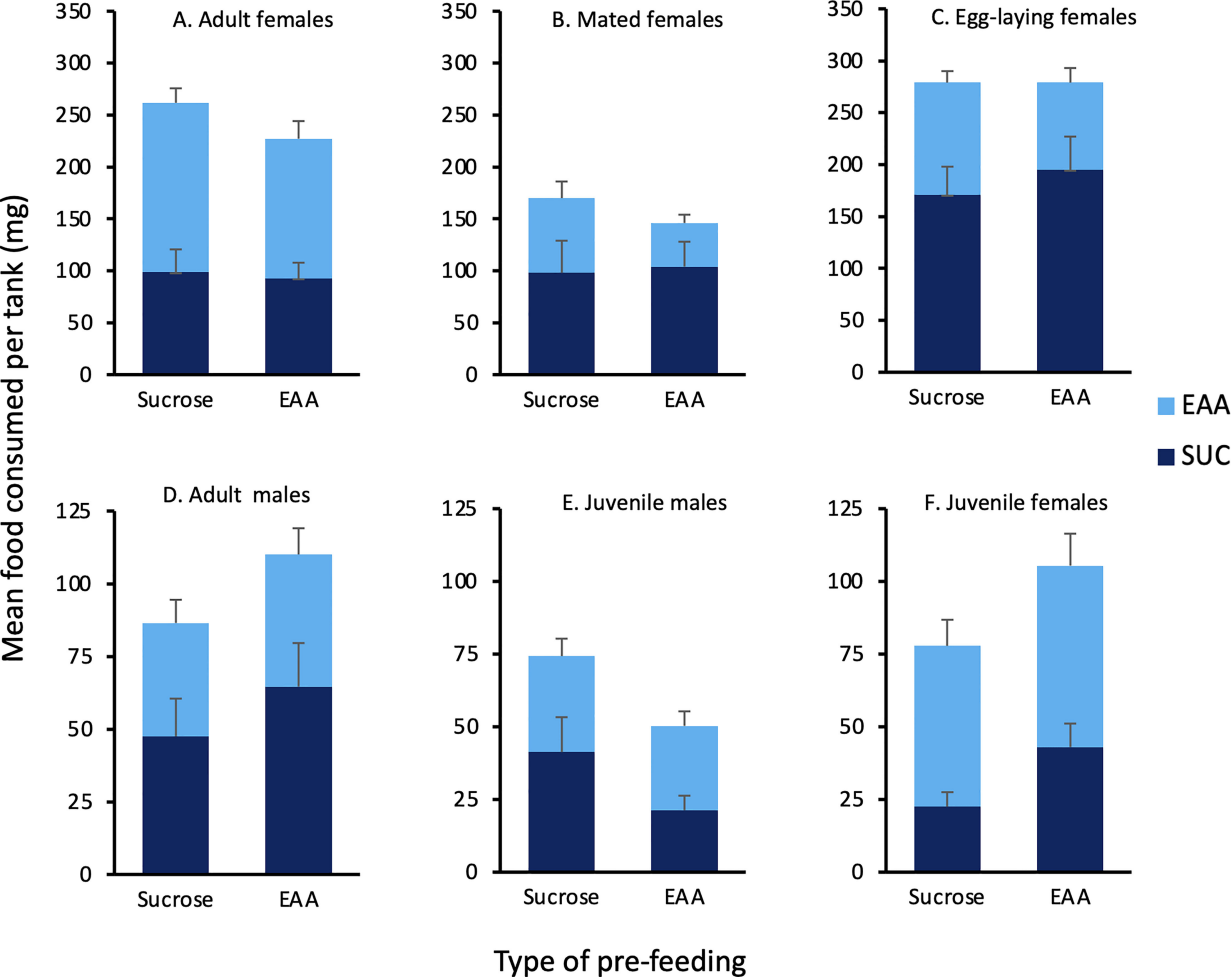
Effects of pre-feeding sucrose or EAA on subsequent consumption of these foods. Bars show the mean amount of sucrose (dark blue) and EAA (light blue) food consumed 24 h after pre-feeding with either sucrose or EAA in the following groups of house crickets: **(A)** Adult virgin females, **(B)** Mated females, **(C)** Egg-laying females, **(D)** Adult males, **(E)** Juvenile males, and **(F)** Juvenile females. See text for statistically significant differences.

The agar test diets were well accepted by the crickets and only four tanks (two juvenile male, one juvenile female, one adult male) were eliminated due to lack of any food consumption. **Figure 2** displays the amount of sucrose and EAA food consumed following pre-feeding with each substance (Experiment 1). In adult virgin females, the intake of EAA was significantly greater than sucrose following both sucrose (Z = -2.073, p = 0.038) and EAA pre-feeding (Z = -2.668, p = 0.008). In juvenile females, the intake of EAA was significantly greater than sucrose following pre-feeding with sucrose (Z = -2.366, p = 0.018). In contrast to these results, mated females significantly preferred sucrose following EAA pre-feeding (Z = -2.395, p = 0.017) and females that produced eggs significantly preferred sucrose following pre-feeding with EAA (Z = -2.803. p = 0.005) and sucrose (Z = -2.075, p = 0.035). Other within group comparisons found insignificant differences in sucrose and EAA intake regardless of preparation. Data on feeding behavior was consistent with the preferences noted above (**Table 1**). A preliminary analysis of 20 randomly selected trials found that most (*M* = 77%) feeding behavior occurred during the first hour and hence our subsequent analysis focused on this time period. Adult virgin females spent significantly more time feeding on EAA disks compared to sucrose disks after both EAA and sucrose pre-feeding, and visited EAA disks more times after EAA pre-feeding. Mated females spent significantly more time feeding on sucrose disks compared to EAA disks after EAA pre-feeding and visited sucrose disks more; results were similar for mated females allowed to lay eggs (**Table 1**). Despite significant differences in feeding time, visits, and food intake, each food was sampled multiple times suggesting that crickets chose to consume more sucrose or EAA after sampling both food types.

**Table 1:** Effects of pre-feeding sucrose or EAA on behavioral responses to each food type in adult virgin female, mated female, post-egg laying female (Eggs), and adult male crickets.

		EAA feeding		SUC feeding				EAA visits		SUC visits			
Group/pre-feed	*N*	*M*	*Mdn*	*M*	*Mdn*	*Z*	*p*	*M*	*Mdn*	*M*	*Mdn*	*Z*	*p*
Virgin♀/EAA	9	1096 ±196	1285	702±148	790	-2.547	.011	15±2.3	17	12±2.1	15	-2.257	.024
Virgin♀/SUC	9	1336 ±177	1249	773±191	446	-2.073	.038	18±3.1	14	15±2.3	12	-1.628	.103
Mated ♀/EAA	10	227±73	156	1132±328	765	-2.803	.005	12±2.5	10.5	16±3.2	16.5	-1.995	.046
Mated ♀/SUC	9	405±62	441	1091±597	303	-415	.678	13±2.0	16	13±2.4	15	-216	.829
Eggs ♀/EAA	10	640±108	641	2419±346	2027	-2.803	.005	20±3.1	18	24±3.9	21	-2.377	.017
Eggs ♀/SUC	9	936±97	930	1768±231	1771	-2.547	.011	17±3.0	17	16±2.1	17	-.354	.723
Adult ♂/EAA	8	301±63	293	676±178	496	-1.680	.093	8±1.5	6.5	12±2.8	9.5	-2.371	.018
Adult ♂/SUC	8	305±98	149	333±81	285.5	-.140	.889	10±1.8	8.5	12±1.9	11.5	-845	.398

EAA feeding, mean (± SE) and median time (sec) crickets spent with mouthparts in contact with EAA food disk; SUC feeding, mean (+± SE) and median time (sec) crickets spent with mouthparts in contact with sucrose food disk; EAA visits, mean (+ SE) and median number of times crickets visited the EAA food disk; SUC visits, mean (± SE) and median number of times crickets visited the EAA food disk.

**Figure 3:**
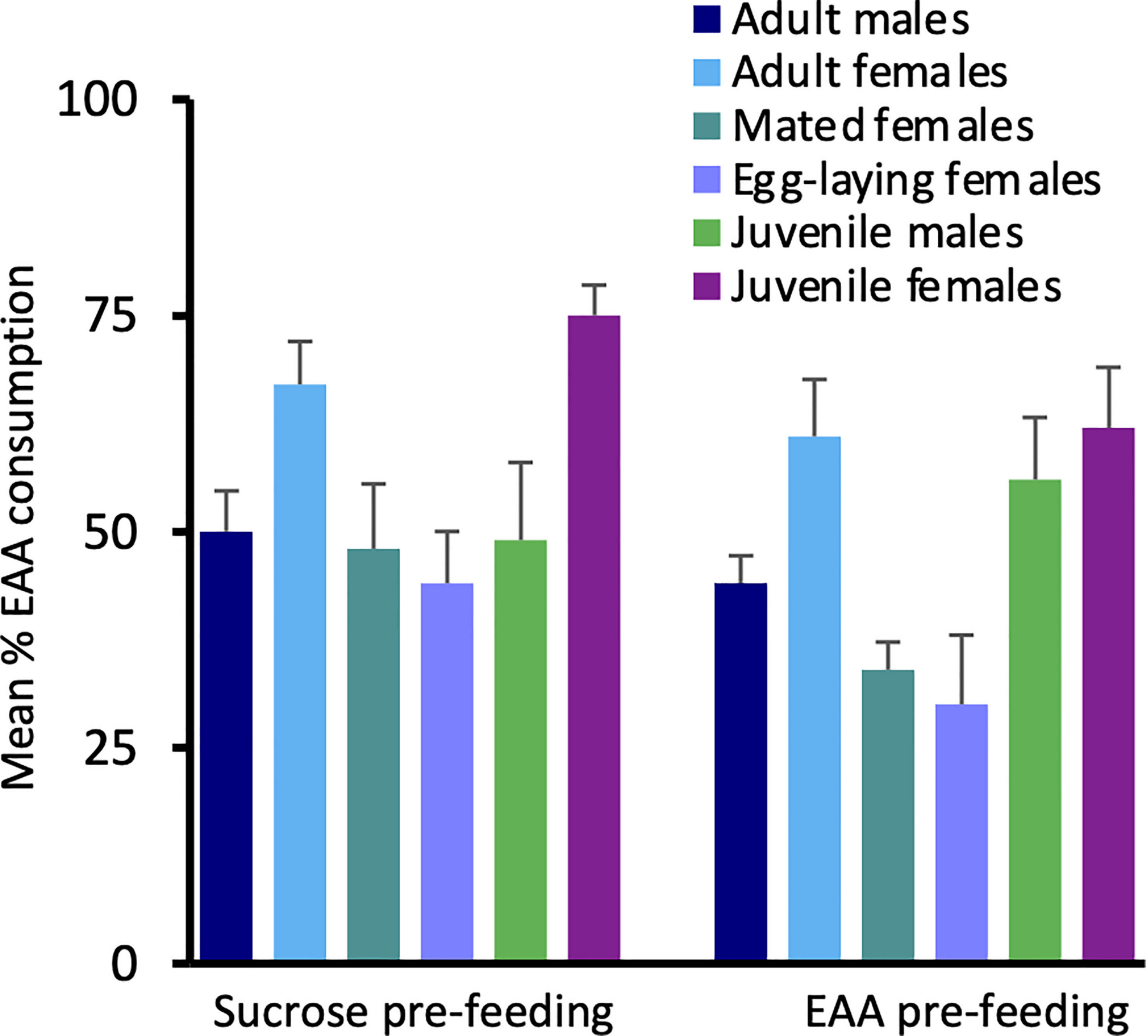
Effects of pre-feeding sucrose or EAA on the percent of EAA food consumed relative to total food consumption. Bars show percent EAA consumption in adult male, adult virgin female, mated female, egg-laying female, juvenile male, and juvenile female house crickets. See text for statistically significant differences.

To examine EAA intake across groups, we compared the percent of EAA food consumed relative to total food intake in the six test groups (**Figure 3**). Following sucrose pre-feeding, there was no significant difference among groups in the percent of EAA food consumed (*H*(5) = 10.61, *p* = 0.06), though intake tended to be greater in adult and juvenile females. Following EAA pre-feeding, the percent of EAA intake differed significantly among groups (H(5) = 20.281, *p* = 0.001). Dunn-Bonferroni pairwise comparisons indicated that mated females post-egg laying consumed significantly less EAA food compared to both adult (*p* = 0.024) and juvenile females (*p* = 0.005). These data combined with the previous comparisons indicate that females differentiate between sucrose and EAA, and favor EAA consumption over sucrose prior to mating. However, this preference reverses after mating and egg-laying. Under the conditions of Experiment 1, juvenile and adult males did not significantly differ in sucrose and EAA food intake though both groups tended to prefer sucrose.

**Figure 4:**
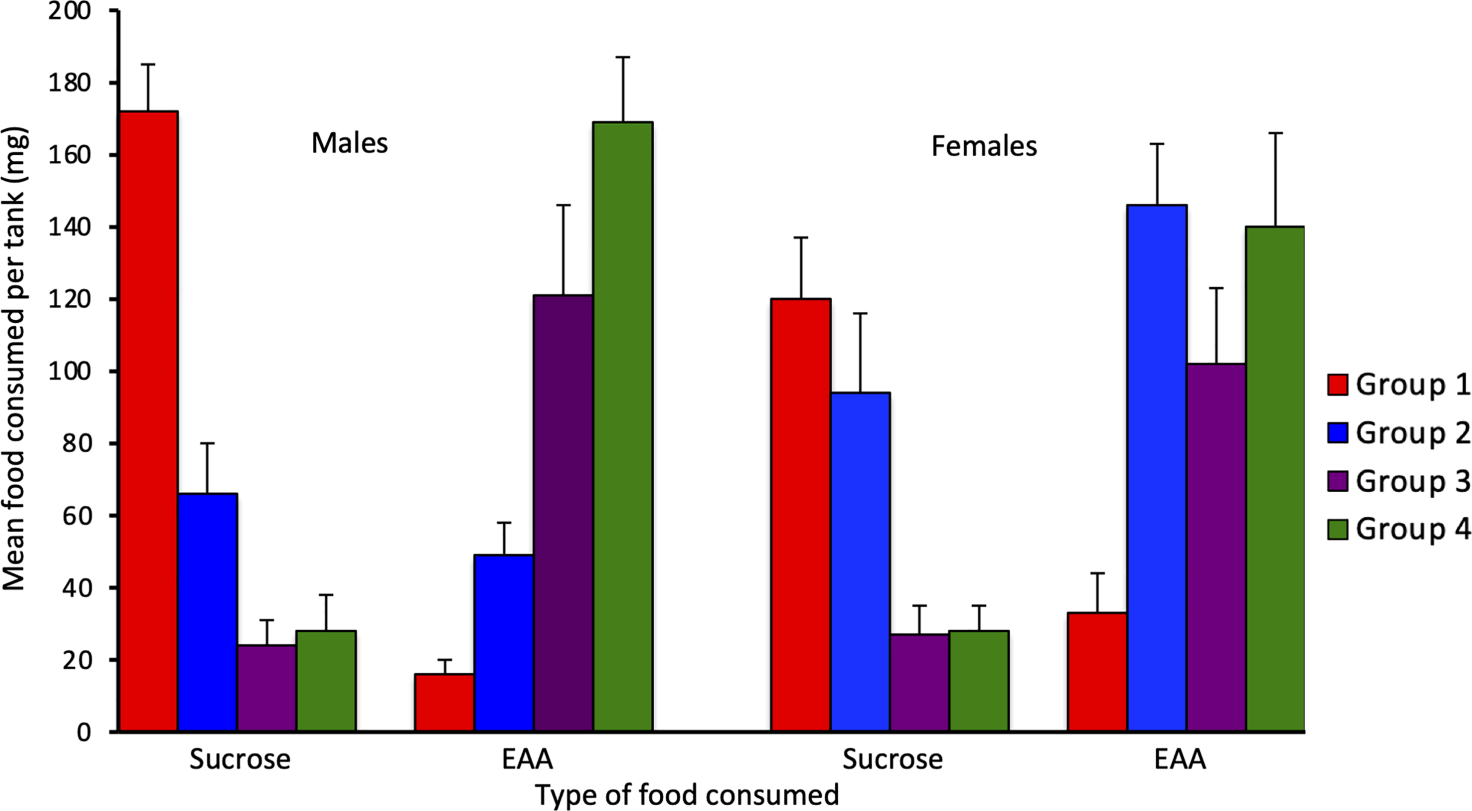
Effects of extended amino acid deprivation in adult male and adult virgin female crickets. Bars show the mean amount of sucrose and EAA consumed in Group 1 (24 h fast only), Group 2 (fed only sucrose for 48 h), Group 3 (fed only sucrose for 72 h) and Group 4 (fed only sucrose for six days). See text for statistically significant differences.

**Figure 5:**
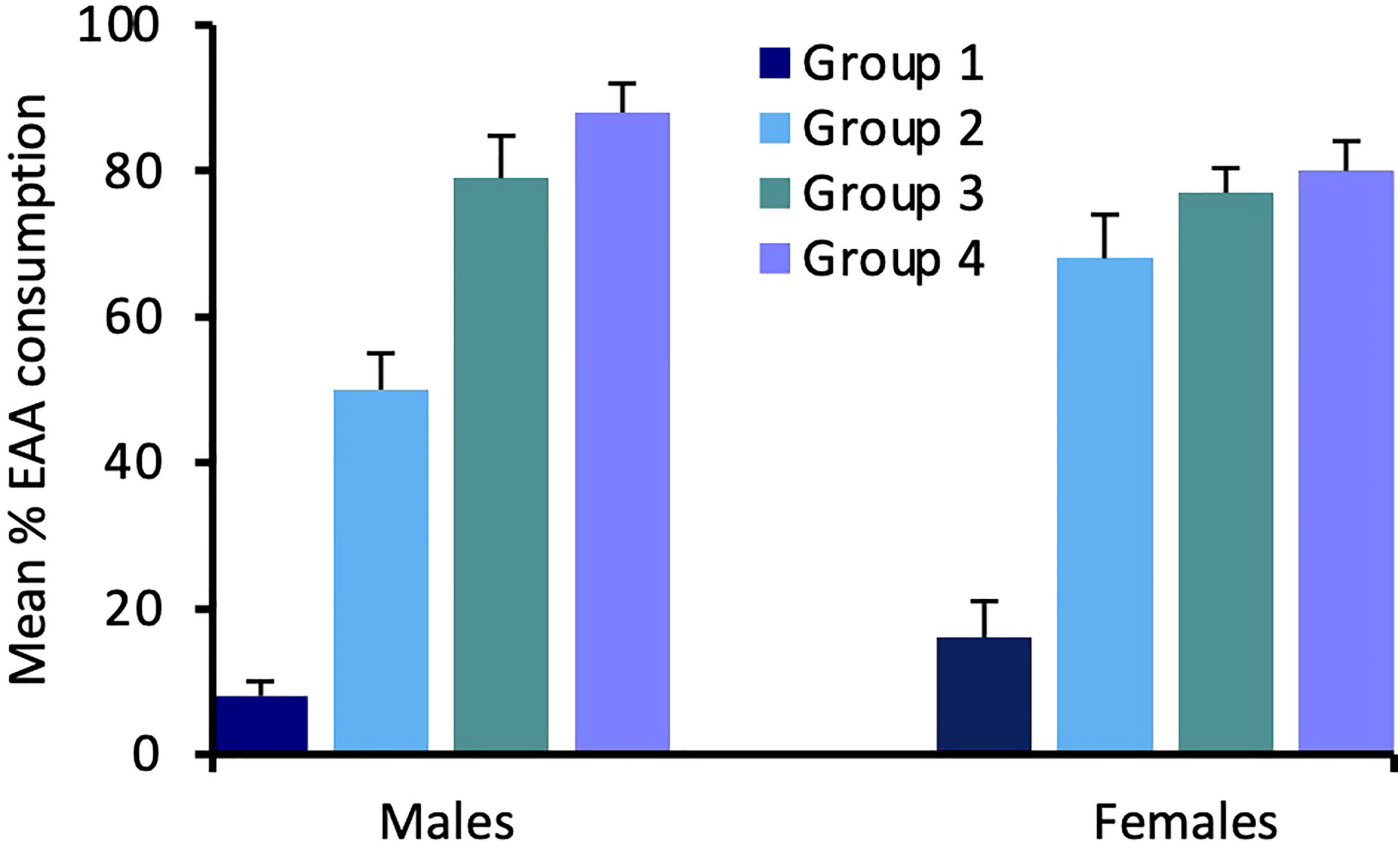
Effects of extended amino acid deprivation on the percent of EAA food consumed relative to total food consumption. Bars show percent EAA consumption in adult male and female crickets in Group 1 (24 h fast only), Group 2 (fed only sucrose for 48 h), Group 3 (fed only sucrose for 72 h) and Group 4 (fed only sucrose for six days). See text for statistically significant differences.

For Experiment 2, consumption of EAA in adult males differed significantly among the four groups tested after 24 h, 48 h, 72 h, and six days of EAA deprivation (H(3) = 16.9, *p* = 0.001; **Figure 4**). We used Dunn-Bonferroni pairwise tests to compare nutrient intake among groups and found that, compared to Group 1 (24 h EAA deprivation), EAA consumption increased significantly in Group 3 (*p* = 0.039) and Group 4 (*p* = 0.001), but not Group 2 (*p* = 0.465). A concomitant decrease in sucrose intake occurred across the four different EAA deprivation periods (H(3) = 19.3, *p* = 0.001). Dunn-Bonferroni pairwise tests indicated that, compared to Group 1, sucrose consumption decreased significantly in Group 2 (*p* = 0.04), Group 3 (*p* = 0.001), and Group 4 (*p* = 0.001). Because the amount of food consumed varied among groups, we also considered the percent of EAA food consumed relative to total food intake in the four groups (**Figure 5**). This analysis confirmed the finding that EEA consumption differed among groups (H(3) = 23.8, *p* = 0.001) and, compared to Group 1, was significantly increased in Groups 3 (*p* = 0.001) and 4 (*p* = 0.001), but not Group 2 (*p* = 0.164). These data indicate that male crickets differentiate between sucrose and EAA, and after 72 h of EAA deprivation their preference switches from sucrose to EAA.

In adult females, consumption of EAA (H(3) = 13.9, *p* = 0.003) and sucrose (H(3) = 14.6, *p* = 0.002) also differed significantly among the four groups (**Figure 4**). For EAA consumption, Dunn-Bonferroni tests indicated that, compared to Group 1, EAA consumption increased significantly in Group 2 (*p* = 0.003) and Group 4 (p = 0.027), but not Group 3 (p = 0.357). For sucrose, compared to Group 1, consumption decreased in Group 3 (*p* = 0.011) and Group 4 (*p* = 0.016), but not Group 2 (*p* = 0.786). A comparison of the percent of EAA food consumed relative to total food intake revealed significant differences among groups (H(3) = 18.3, *p* = 0.001) and significant increases in EAA intake in Groups 2 (*p* = 0.032), 3 (*p* = 0.003), and 4 (*p* = 0.001), relative to Group 1 (**Figure 5**). These data support the previous finding that females differentiate between sucrose and EAA and they switch their preference from sucrose to EAA after 48 h of EAA deprivation.

**Figure 6:**
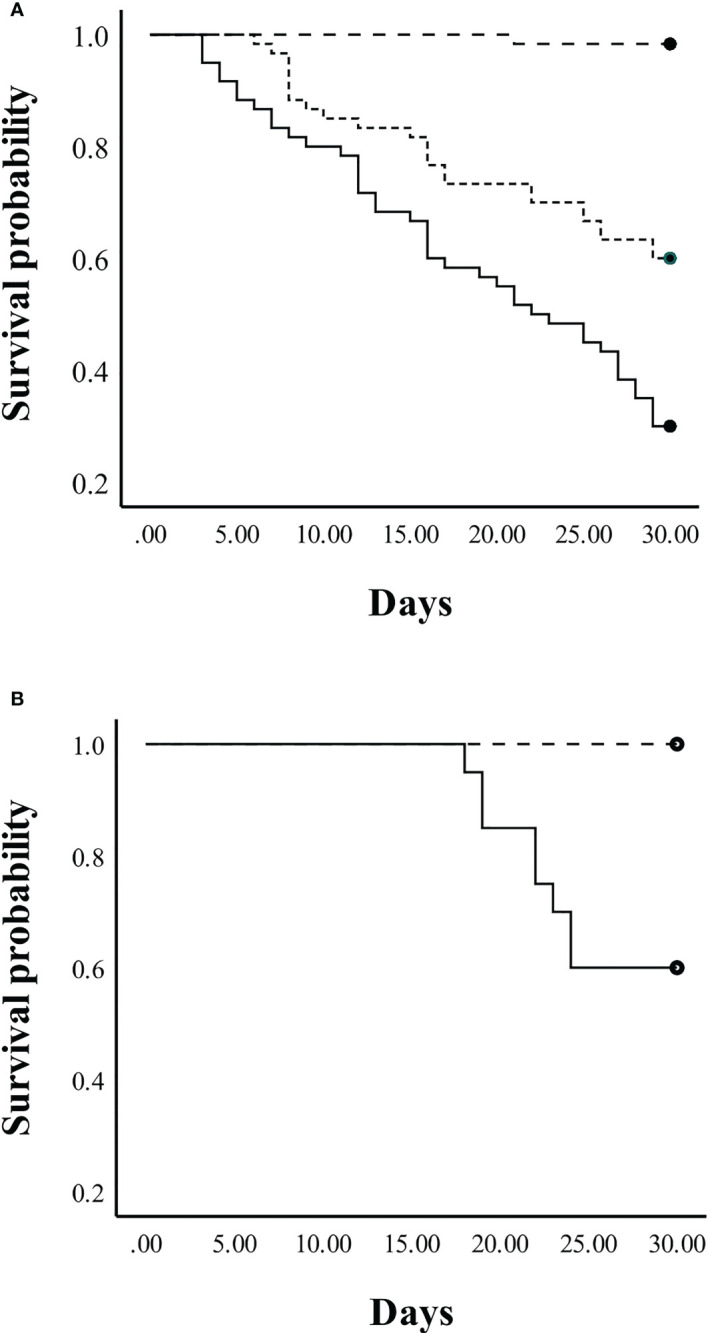
Survival probability of juvenile crickets fed a control diet (dashed line) or a holidic diet with EAA (dotted line) or without EAA (solid line) for 30 days. **(A)** Survival probability of juvenile crickets housed in same-sex community tanks. **(B)** Survival probability of juvenile crickets held in isolate tanks. See text for statistically significant differences.

In Experiment 3, the overall survival of juvenile crickets in community tanks differed significantly among the three diets (log-rank test: *X^2^* = 61.4, *p* < 0.001, **Figure 6A**). Pairwise log-rank comparisons indicated that survival in the control diet differed significantly from the EAA diet (*X^2^* = 26.8, *p* < 0.001) and the No EAA diet (*X^2^* = 63.9, *p* < 0.001); also, the EAA and No EAA diets differed significantly in juvenile survival (*X^2^* = 10.5, *p* = 0.001). Crickets fed the control diet had a 98% survival rate, an average increase in weight of 320 mg/cricket, and 78% (47 insects) had molted to adulthood by the end of the 30-day period. Crickets fed the artificial diet with EAA or No EAA had survival rates of 60% and 30%, respectively. However, growth was severely restricted in both groups (average weight increase in EAA group: 21 mg/cricket; No EAA group: 14 mg/cricket) and no insects molted to adulthood. These data indicate that, while both artificial diets limited growth, access to EAA significantly enhanced survival. For juvenile crickets held individually, survival was significantly greater in the control group compared to the crickets in the No EAA group (log rank test: *X^2^* = 9.8, *p* < 0.002, **Figure 6B**). All juveniles fed the control diet survived and molted to adulthood during the 30-day period, whereas 60% of the crickets fed the No EAA diet survived and growth was restricted (average weight increase over 30 days was 15 mg/cricket). These data confirm the superior performance of juveniles fed control food and suggest that reduced survivorship in the community tanks fed the No EAA diet may have been due in part to greater levels of cannibalism. All adult crickets of both sexes survived for 30 days when fed the control, EAA, or No EAA diet, indicating that the three diets were able to sustain life for this time period.

## Discussion

The present results contribute several novel findings about nutrient selection in the house cricket. First, we found that house crickets can distinguish EAA from sucrose and that selection of these two nutrients is affected by prior feeding experience. We also found that age and reproductive state affected food choice, with juvenile and virgin females showing a greater preference for EAA than juvenile or adult males. Contrary to previous studies, mated females preferred sucrose over EAA both prior to and after egg laying. We also found that crickets of both sexes increased their intake of EAA when exposed to longer periods of deprivation, suggesting that they engage in compensatory feeding on these nutrients. Finally, we found that growth was severely limited in juveniles fed a diet lacking all amino acids. However, many juveniles and all adults survived on this diet for 30 days indicating that they possess considerable resilience to dietary restriction.

Our results support previous studies which have found that insects respond behaviorally to free amino acids when making feeding decisions. These chemicals may serve as attractants or deterrents in nectar feeders, allowing insects to gain needed nutrients while avoiding excessive amino acid intake (20, 22, 23). In *Drosophila*, adaptive responses to amino acids may likewise allow larvae (39) and adults to acquire an appropriate balance of these nutrients and respond to deprivation with enhanced amino acid preference. House crickets are highly generalized feeders and are likely to occur in areas where intensive farming creates environments with nutritional imbalances (40). Hence, they may encounter foods that vary in the quantity and quality of amino acids or where abundant food sources lack EAA. Dietary challenges may be exacerbated by the tendency of house crickets to aggregate (41), causing social stress and competition for food (42, 43). Hence the ability to detect EAA and compensate for deprivation is likely to be especially adaptive in these insects. The site of amino acid perception has not yet been established in crickets, but in other insects individual amino acids can be detected by taste sensilla on the legs (18, 44) and labellum (45, 46). Recent studies have also identified molecular receptors for amino acids in *Drosophila* including the odorant binding protein OBP19b (47), the ionotropic receptor Ir76b (18, 39), and several additional ionotropic and gustatory receptors (46). Comparative research is needed to determine if these mechanisms are conserved across other insect species.

In experiment 1, we found that after 48 h EAA deprivation, juvenile and adult virgin females chose to consume more EAA than sucrose; however, juvenile and adult males did not display a preference for EAA under the same conditions. When protein is limited, free EAA are a source of required nutrients and the attraction of females to EAA is consistent with the tendency of female crickets to consume more protein than males (31). Oogenesis in house crickets begins on day three following adult ecdysis and oviducts are full by day 15 (48), the period of time during which we tested virgin females. Hence the need to produce eggs may drive EAA appetite in females just prior to and after the adult ecdysis. The preference for sucrose after mating and egg laying in female house crickets contrasts with findings in *Drosophila* (15, 33) and two-spotted crickets (34) in which protein ingestion increased after mating. Our results possibly reflect motivation to replenish energy *via* carbohydrate feeding immediately following periods of heightened physical activity and decreased food intake which occur during mating and egg laying (48). In future studies, it would be useful to examine food preferences throughout adulthood to better understand the associations between protein or EAA ingestion and oogenesis, mating, and oviposition.

In male crickets, sucrose pre-feeding for 72 h and 6 days induced a switch from sucrose to EAA preference (Experiment 2). Virgin females also consumed more sucrose after a 24 h fast, but consistent with the results of Experiment 1, preferred EAA after 48 h deprivation and one sucrose pre-feeding. These results indicate that both sexes perceive and compensate for EAA deficiency. However, under our experimental conditions, house crickets did so only after a relatively long period of deprivation whereas some other species displayed compensatory selection of carbohydrate and protein after dietary restrictions lasting only a few hours (12, 13). The results of the survival study (Experiment 3) are interesting in this context since they indicate that adult crickets displayed resilience in the face of EAA deprivation given that 100% of adults of both sexes survived for 30 days on sucrose alone. This suggests that, as an omnivorous and opportunistic feeder, adult house crickets have evolved to tolerate temporary nutritional imbalances and may not be under strong selective pressure to very rapidly compensate for dips in EAA availability. The situation is different for juvenile stages since we found that growth and survival were significantly impacted by lack of EAA in 4^th^ instar nymphs. We did not assess dietary choice in early cricket instars, but because optimal growth depends on EAA availability it is possible that a compensatory response to EAA deprivation would occur sooner in juveniles relative to adults.

Many additional discoveries remain to be made about cricket diet selection. Further investigations of feeding behavior at different life stages would contribute additional information on the adjustments insects make to optimize nutritional balance as they mature and reproduce (3). In addition, other major behavioral events such as aggressive interactions and courtship singing may trigger changes in nutrient selection, a possibility that remains to be examined in crickets and other species. Another topic of interest concerns the perception and selection of individual amino acids. Previous work has shown that some insects distinguish among single amino acids; for example, *Drosophila* larvae are most attracted aspartic acid and avoid leucine (19, 39), and honey bees are more attracted to essential amino acids, especially phenylalanine, compared to nonessential amino acids (23). Such information is not yet available in crickets and would be useful for comparative purposes and as a foundation for future physiological studies. The latter are important since the neural mechanisms used by crickets to sense amino acids, both externally and internally, are not yet elucidated. Finally, amino acid appetite may differ between insects from inbred laboratory colonies and their wild counterparts. For example, in the laboratory locusts regulate nutrient intake in a consistent manner, whereas wild locusts display considerable plasticity in their feeding behavior across generations and seasons (49). Also, gut microbial communities differ between wild crickets and those confined and fed a laboratory diet, a factor that may affect how amino acids are synthesized and degraded (50, 51). Our study examined amino acid appetite in laboratory animals fed a commercial cricket diet and more general conclusions could be drawn by extending the experiments to wild populations.

## Data availability statement

The raw data supporting the conclusions of this article will be made available by the authors, without undue reservation.

## Author contributions

AT designed the study, ran experiments, analyzed data, and wrote the original draft of the manuscript. EV, LJ, SH, MP, and MS contributed to data collection and analysis. All authors contributed to the article and approved the submitted version.
